# Supplementary material for: In Vitro Activity of Cefiderocol Against Multi-Drug-Resistant Gram-Negative Clinical Isolates in Romania
Source: Antibiotics (Basel). 2025 Nov 5;14(11):1113. doi: 10.3390/antibiotics14111113 (PMC12649249; doi:10.3390/antibiotics14111113)
Supplement: Supplementary file 1 [file antibiotics-14-01113-s001.zip › antibiotics-3937772-supplementary.pdf]

Supplementary Table S1. **EUCAST and CLSI current breakpoints for cefiderocol susceptibility testing**

Organism	MIC breakpoint (mg/L) (EUCAST)		Growth inhibition zone (mm) EUCAST			MIC breakpoint (mg/L) (CLSI)			Growth inhibition zone (mm) CLSI		
	**S ≤**	**R >**	**S≥**	**ATU**	**R <**	**S≤**	**I**	**R>**	**S≥**	**I**	**R<**
Enterobacterales	2	2	23	21-23	23	4	8	16	16	9-15	8
Pseudomonas aeruginosa	2	2	22	21-22	22	4	8	16	18	13-17	12
Stenotrophomonas maltophilia	-	-	20	-	-	1	-	-	15	-	-

Notes

EUCAST recommendations

**For Acinetobacter baumannii there are no available data to establish the breakpoints. On the basis of pharmacokinetic and pharmacodynamic activity of Cefiderocol, a diameter of inhibition ≥ 17 mm corresponds to a MIC ≤* 2 mg/L.

CLSI recommendations

For Acinetobacter baumannii complex, there are insufficient data to establish all susceptibility values. However, on the basis of pharmacokinetic and pharmacodynamic activity of Cefiderocol on these strains, a diameter of inhibition ≥ 15 *mm has been considered to represent susceptibility to this antibiotic.*

***The zone diameters with a value <= 14 mm should not be reported without performing an MIC test as these diameters occur with both resistant, intermediate and susceptible strains*

ATU – Area of Technical Uncertainty
